# Preclinical efficacy and rationale of targeting lysine280‐acetylated tau for Alzheimer’s disease therapy with ADEL‐Y01, humanized monoclonal antibody

**DOI:** 10.1002/alz.088589

**Published:** 2025-01-09

**Authors:** Min‐Seok Kim, Ha‐Lim Song, Na‐Young Kim, Yeon‐Seon Mun, Dong‐Hou Kim, Seung‐Yong Yoon

**Affiliations:** ^1^ ADEL Institute of Science & Technology (AIST), ADEL, Inc., Seoul Korea, Republic of (South); ^2^ Asan Medical Center, University of Ulsan College of Medicine, Seoul Korea, Republic of (South)

## Abstract

**Background:**

The spatiotemporal pattern of the spread of pathologically modified tau through brain regions in Alzheimer’s disease (AD) can be explained by prion‐like cell‐to‐cell seeding and propagation of misfolded tau aggregates. Hence, to develop targeted therapeutic antibodies, it is important to identify the seeding‐ and propagation‐competent tau species. The hexapeptide ^275^VQIINK^280^ of tau is a critical region for tau aggregation, and K280 is acetylated in various tauopathies including AD. However, the mechanism that links tau acetylated on lysine 280 (tau‐acK280) to subsequent progression to neurodegenerative disease remains unclear. Here, we demonstrate that tau‐acK280 is critical for tau propagation processes including secretion, aggregation, and seeding.

**Method:**

We developed an antibody, Y01, that specifically targets tau‐acK280, tested its efficacy in vitro and in vivo tauopathy models, and solved the crystal structure of Y01 in complex with an acK280 peptide.

**Result:**

Upon interaction with acetylated tau aggregates, Y01 prevented tauopathy progression and increased neuronal viability in neuron cultures and in tau transgenic mice through antibody‐mediated neutralization and phagocytosis, respectively. Crystal structure confirmed that Y01 directly recognizes acK280 and the surrounding residues.

**Conclusion:**

Based on our observations that tau‐acK280 is a core species involved in seeding and propagation activities, the Y01 antibody that specifically recognizes acK280 represents a promising therapeutic candidate for AD and other neurodegenerative diseases associated with tauopathy. ADEL‐Y01 is currently undergoing a phase‐1 clinical trial in the United States through a collaborative effort between ADEL and Oscotec.

